# Translating genomics research into control of tuberculosis: lessons learned and future prospects

**DOI:** 10.1186/s13059-014-0514-z

**Published:** 2014-11-07

**Authors:** Digby F Warner, Valerie Mizrahi

**Affiliations:** MRC/NHLS/UCT Molecular Mycobacteriology Research Unit, Institute of Infectious Disease and Molecular Medicine, Faculty of Health Sciences, University of Cape Town, Anzio Road, Observatory, Cape Town, 7925 South Africa; DST/NRF Centre of Excellence for Biomedical TB Research, Institute of Infectious Disease and Molecular Medicine, University of Cape Town, Anzio Road, Observatory, Cape Town, 7925 South Africa; Division of Medical Microbiology, Department of Clinical Laboratory Sciences, University of Cape Town, Anzio Road, Observatory, Cape Town, 7925 South Africa

## Abstract

Genomics research has enabled crucial insights into the adaptive evolution of *Mycobacterium tuberculosis* as an obligate human pathogen. Here, we highlight major recent advances and evaluate the potential for genomics approaches to inform tuberculosis control efforts in high-burden settings.

## Introduction

Tuberculosis (TB) is a leading cause of death as a result of an infectious bacterial agent, claiming 1.4 million lives each year [1]. With an estimated global burden of 8.7 million incident cases per annum, TB remains a major public health threat. In high-burden regions such as sub-Saharan Africa, the TB epidemic is exacerbated by co-morbidities, including HIV and diabetes, as well as demographic, socioeconomic, and programmatic factors [2]. The magnitude of the TB problem has been further amplified by the evolution and global spread of strains of *Mycobacterium tuberculosis* that are resistant to conventional first- and second-line antitubercular drugs. Of particular concern, drug resistance is worsening, having progressed from multi-drug resistant (MDR), to extensively drug resistant (XDR), to ‘functionally untreatable’ [3] TB - that is, disease for which no therapeutic options remain. This progression has led to calls for ‘visionary political leadership’ [4] and ‘increased funding to sustain global control efforts, research and advocacy’ [3]. In order to reach the aspirational goal of global TB elimination by 2050, TB incidence will need to be reduced by approximately 16% each year for the next 40 years. In spite of recent gains in the battle against TB, the current rate of decline in TB incidence of 2% per annum falls far short of this target [5]. This alarming situation underscores the urgent need for new tools to control this devastating disease.

Fundamental TB research poses very specific practical and financial challenges. As an infectious pathogen, *M. tuberculosis* can only be manipulated in purpose-built biosafety level 3 containment laboratories by specialist personnel. The construction and maintenance of such facilities requires significant financial investment; moreover, the running costs necessary to ensure continued compliance with the stringent safety regulations are high, and are incurred in addition to standard laboratory operating expenses. From a practical perspective, *M. tuberculosis* is an intractable experimental subject: the bacillus is notorious for its slow growth rate *in vitro* and for its tendency to form aggregates in liquid media. As a result, experiments are technically demanding, long in duration and prone to contamination. The combined effect, therefore, is that the achievement of definitive results can be very slow.

Even more challenging are the scientific problems posed by the natural lifecycle of *M. tuberculosis* as an obligate human pathogen. By definition, all experiments conducted outside infected individuals - whether *in vitro* or *in vivo* - are performed in model systems that have varying capacities to recapitulate specific aspects of the host-pathogen interaction. Although advances in experimental mycobacteriology have provided key insights into the metabolic and regulatory pathways that are critical for bacillary survival and pathogenesis, it remains extremely difficult to determine the precise physiological status of tubercle bacilli during different stages of infection and in discrete anatomical and cellular (micro)environments. As noted elsewhere [6], an important consequence is that direct investigations of mycobacterial function in the context of the complete biological system - the *M. tuberculosis*-infected host - remain rare. In turn, this means that the barriers to translating the observations from basic research into practical outcomes are sizeable.

**Figure 1:**
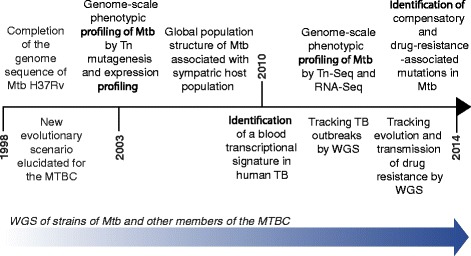
**Timeline of key studies in tuberculosis genomics research.**

The application of genomics and other ’omics technologies in developing a systems biology of TB is central to global efforts towards the development of new vaccines, diagnostics and drugs for TB. The landmark publication in 1998 by Stewart Cole and colleagues [7] of the first genome sequence of a strain of *M. tuberculosis* ushered in a new era in TB research in which genome-scale studies have provided crucial insights into the ancient and modern evolutionary history of *M. tuberculosis*, the genomics of drug resistance, the biology of *M. tuberculosis* as an intracellular pathogen, and the host response to infection with this organism (Figure 1). In this article, we highlight the major advances in TB research that have been enabled by the genomics revolution. We then identify key areas of research and development that will be required in order to harness the full potential of genomics approaches for the control of TB in endemic regions, discuss some of the major challenges and obstacles that will need to be addressed and overcome in this endeavor, and conclude by considering the implications of the lessons learned from TB in the context of other infectious diseases.

## The evolutionary history of *M. tuberculosis*

*M. tuberculosis* is one member of the *M. tuberculosis* complex (MTBC), a collection of phylogenetically linked organisms comprising eight closely related lineages [8] and the outlying *M. canettii* group, in which the so-called ‘smooth tubercle bacilli’ are situated [9]. *M. tuberculosis sensu stricto* from lineages L1 to L4 and L7, together with the *Mycobacterium africanum* lineages L5 and L6, are human-adapted, whereas the L8 lineage - which includes *Mycobacterium bovis* and the TB vaccine strain, BCG (Bacille Calmette Guérin) - contains the animal-adapted pathogens. The recent discovery of chimpanzee and mongoose bacilli [10,11] suggests, however, that there might be much greater diversity within the MTBC. In turn, this implies that existing scenarios for the evolution of the human- and animal-adapted strains might be overly simplistic, and limited by the availability of isolates, especially from wild mammals [11]. Defining the point in time, as opposed to the phylogenetic position, at which MTBC strains originated from a last common ancestor has proven very difficult [8,12,13]; nevertheless, both comparative genomics and bioarcheological evidence support the extended co-evolution of *M. tuberculosis* with its obligate host [14]. In turn, this implies the evolution of a conserved host-pathogen interaction that enables repetitive cycles of infection, disease, and transmission while accommodating bacillary adaptation to major human demographic shifts. Although not conclusive, evidence of selective pressure on specific mycobacterial antigens provides some support for this idea [15], as does the observation that diverse *M. tuberculosis* strains engage a core transcriptional response following macrophage infection, while exhibiting hallmarks of lineage-specific adaptation to geographically varied host populations [16]. Notably, the interaction between a particular locally adapted *M. tuberculosis* strain and its corresponding geographically matched host appears to depend on a functional immune response: these sympatric interactions are disrupted by HIV co-infection [17].

Unlike most other bacterial pathogens, a defining characteristic of *M. tuberculosis* is its reliance on chromosomal rearrangements and mutations as drivers of genomic evolution [14]. Horizontal gene transfer (HGT) certainly played an important role in the evolution of *M. tuberculosis* as a human pathogen [14,18,19]; however, despite the proposal that ongoing recombination provides a source of genetic variation [20], there is very little evidence in support of a role for HGT in the modern evolution of this organism [21]. This feature is likely to result from the ecological isolation of the bacillus as an obligate pathogen that primarily targets the host pulmonary and lymphatic system [22], as well as from the severe bottlenecks imposed by aerosol-dependent transmission from infectious individual to naïve recipient [23].

## Insights from diversity between lineages of the MTBC

As noted above, the MTBC comprises eight closely related lineages [8] which can be distinguished according to a lineage-defining single nucleotide polymorphism (SNP) ‘barcode’ [24]. Until very recently, the functional consequences of almost all of the differentiating SNPs remained completely unexplored. In an important study illustrating the power of integrating ’omics with mycobacterial genetics and chemical biology in experimental models of TB infection, Christophe Guilhot, Roland Brosch and colleagues demonstrated that SNPs which are conserved in animal-adapted and *M. africanum* L6 strains are less transmissible and virulent in humans than *M. tuberculosis sensu stricto* [25]. Guided by insights from comparative genomics, these researchers homed in on three separate SNPs that map to the promoter region of *phoP* and codon 71 of *phoR*, genes which encode a two-component system previously implicated in the virulence and immunogenicity of *M. tuberculosis*. This system regulates the synthesis and export of virulence factors that include the major secreted antigen, ESAT-6, as well as polyacyltrehalose (PAT) lipids and sulfolipids (SLs). By transferring any of three alleles - *M. tuberculosis phoPR*, *M. bovis phoPR*, or a chimeric *phoPR* allele in which the *phoP* (promoter) and *phoR* (coding region) SNPs were split - into a *phoPR* null mutant of *M. tuberculosis*, the authors demonstrated that the *M. bovis phoR* allele is associated with impaired expression of the PhoPR regulon. The *M. bovis phoPR* allele was also found to impact negatively on mycobacterial virulence in human macrophage and mouse models of infection.

Armed with these data associating genotype with phenotype, the authors then set out to characterize the PhoPR system in a set of wild-type animal-adapted and *M. africanum* L6 strains, as well as in matched derivatives harboring the *M. tuberculosis phoPR* allele.

The levels of PAT and SL lipid families were comparatively low in the wild-type strains but markedly higher in their counterparts that carry *M. tuberculosis phoPR*, but the same was not true for ESAT-6, which was secreted at comparable levels in the wild-type and recombinant pairs. The animal-adapted and *M. africanum* L6 strains therefore appear to have acquired compensatory mutations that ameliorate the defect in ESAT-6 production caused by the SNPs in *phoPR*, and so partially restore virulence. In a further twist, convincing evidence was obtained that implicates the insertion of an IS*6110* element upstream of *phoPR* in the hypervirulent phenotype of *M. bovis* B - an MDR isolate of *M. bovis* responsible for an outbreak of TB in Spain [26] - resulting from suppression of the functional deficiencies of the *M. bovis phoPR* allele.

Importantly, this study reinforces the need to sequence additional panels of clinical *M. tuberculosis* isolates as well as other MTBC strains [18] to identify evidence of convergent evolution of functions that might impact bacillary pathogenesis. In contrast to the *M. canettii* group, whose larger genomes have been shaped by extensive inter-strain recombination and horizontal transfer [9,18], the population structure of the MTBC is clonal. It is likely that this clonal restriction, which is evident in the identification of 2,400 SNPs (at most) in a 4.4 Mb MTBC genome, reflects the combined selective pressure of obligate pathogenesis, as well as the close association of MTBC with their natural hosts. In addition, the impact on apparent diversity of strain sampling and laboratory propagation remains unclear. For this reason, the recent use of shotgun metagenomics in clinical TB samples [27] is encouraging, as it suggests that ‘culture-free’ techniques might enable key insights into the mycobacterial population structure in specific anatomical compartments, while avoiding the biases inherent in existing sample-collection techniques.

## Understanding the genomics of TB drug resistance

In no other area of TB research has the impact of genomics been more profound than in establishing the mechanisms that enable the resistance of *M. tuberculosis* to TB drugs. Like analogous research on other bacterial pathogens, elucidation of the genetic basis of resistance of *M. tuberculosis* to the first-line drugs for the treatment of TB - isoniazid, rifampicin, ethambutol and pyrazinamide - pre-dated the introduction of routine whole-genome sequencing (WGS) of resistant mutants [28]. The discovery that the majority of rifampicin resistance-conferring mutations found in clinical isolates map to an 81-bp region within the *rpoB* gene enabled the development and implementation of the new molecular diagnostic, Xpert MTB/RIF. This test allows for rapid identification of *M. tuberculosis* within clinical specimens and simultaneous identification of rifampicin resistance - a key genetic marker of MDR-TB [29,30]. Assessing the medical, public health and economic impacts of this potentially ‘game-changing’ technology [31] is the subject of intense investigation in South Africa, where an ambitious program to roll-out Xpert MTB/RIF nationally is underway [32].

More recently, WGS has been used to analyze strains of *M. tuberculosis* with varying drug susceptibility profiles from collections of clinical isolates, as well as drug-resistant mutants isolated in the laboratory [33]. In addition to identifying both canonical resistance-conferring mutations and compensatory mutations, the comparative genomic analyses of Farhat *et al*. [34] and Zhang *et al.* [35] identified a significant number of new resistance-associated mutations not previously implicated in genetic drug resistance [36]. Their observations suggest that the development of drug resistance in *M. tuberculosis* is a more complex biological phenomenon than previously thought - a notion consistent with emerging trends in other areas of anti-microbial drug resistance [37]. However, the impact of these potentially novel resistance-associated mutations on mycobacterial pathogenesis, and their functional contribution to TB drug resistance, is poorly understood. Validation of the association between genotype and phenotype requires transfer of the resistance-associated mutations into a defined genetic background by means of allelic exchange, a laborious and time-consuming exercise not routinely applied in the TB field, even in the case of resistance-conferring mutations [38]. Therefore, although new techniques such as recombineering offer promise of improved throughput for targeted allelic mutagenesis [39], the genetic validation of resistance-linked mutations is likely to remain a significant challenge. Attempts to confirm inferred associations between specific mutations and observed decreases in drug susceptibility are further complicated by the increasing awareness of the significant capacity of mycobacterial populations for phenotypic heterogeneity in the response to applied drugs [40]. In addition, recent evidence of strain-specific transcriptional phenotypes suggests that genetic background might be of crucial importance in determining the functional consequences of specific mutations [41].

In an impressive illustration of the application of WGS in analyzing the genomics of TB drug resistance, Casali *et al*. [42] investigated the mechanisms underlying the evolution and transmission of TB drug resistance in Russia by sequencing 1,000 *M. tuberculosis* isolates collected prospectively from clinical TB patients. Notably, the major Beijing lineage clades in this collection of strains were found to contain combinations of resistance and compensatory mutations that conferred TB drug resistance while retaining fitness and transmissibility. Traditionally, public health strategies to counter the threat of drug-resistant TB have focused almost entirely on programmatic issues; however, in highlighting the importance of (micro)biological factors in the persistence and spread of MDR and XDR strains within a population [42], this study added a disturbing new dimension to an already daunting challenge.

Another area in which WGS analysis of resistant isolates has been applied is in the identification of putative targets of novel anti-mycobacterial agents discovered by screening compound libraries for whole-cell activity against *M. tuberculosis*. This method was successfully used to identify the targets of bedaquiline (the AtpE subunit of ATP synthase), the benzothiazinone BTZ043 (the DprE1 epimerase), and the imidazopyridine amide Q203 (the QcrB subunit of the respiratory cytochrome *bc*_1_ complex) [43]. However, as mutations that compromise drug efficacy frequently map to other resistance-linked genes (such as those encoding efflux pumps) rather than the target, the utility of this method for target identification in *M. tuberculosis* is somewhat limited [39].

## Insights from genome-wide phenotypic profiling of *M. tuberculosis*

**Figure 2:**
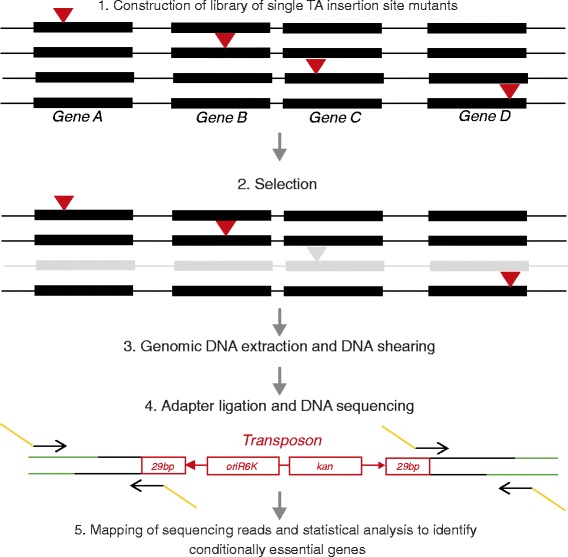
**Transposon sequencing (Tn-seq) methodology.** A Tn library is prepared by infecting *M. tuberculosis* with the temperature-sensitive MycoMarT7 bacteriophage, which results in transposon (Tn) insertion at genomic loci that contain TA sites. The Tn, denoted as an inverted red arrowhead, contains a kanamycin resistance gene (*kan*) that is utilized to select cells that contain a Tn insertion, the *E. coli* oriR6K origin of replication, two outward facing T7 promoters (red arrows in (4)), and 29-bp inverted repeats. Tn insertions that disrupt four genes, A to D, are represented in the library. The library is then subjected to selection under any condition of interest. Tn mutants carrying an insertion in a gene that is essential under that condition will not survive, as illustrated in this schematic by gene C. After selection, genomic DNA is extracted from surviving organisms, sheared, and T-tailed adapters (denoted by green lines) are then ligated to the DNA ends. Adapter-specific and Tn-specific primers with extensions homologous to Illumina sequencing primers (orange lines) are then used for direct sequencing on Illumina platforms. Sequence reads are trimmed at the Tn region, and mapped to the parental strain genome. Genes that have no or few insertions are likely to be important for survival under the selective condition. The schematic is adapted from Zhang *et al*. [58].

As in other fields of microbiology [44], the advent of functional genomics has led to major advances in understanding the biology of *M. tuberculosis* through global phenotypic profiling. This has allowed associations between genotype and phenotype to be uncovered, and has enabled the systematic identification of genes which are required for bacillary growth and survival under conditions that are thought to prevail during human infection. The early application of array-based methods such as transposon site hybridization (TraSH) [45] and signature-tagged mutagenesis [46] provided key insights into the genetic requirements for growth of *M. tuberculosis in vitro* [47,48], in macrophages [49], and in animal tissue [50-54]. Recently, these methods have been superseded by transposon sequencing (Tn-Seq), an example of the numerous ‘multiletter acronym’ or ‘MLA-seq’ applications [55] that have transformed post-genomic research. In the context of TB, these applications have enabled global phenotypic profiling at significantly higher resolution (Figure 2). Tn-Seq has been used to refine the list of genes required for the growth of *M. tuberculosis* under standard *in vitro* conditions, and to identify the genes needed for growth on cholesterol, a critical carbon source during infection [56]. In an exciting new study that elegantly illustrates the power of this approach, Zhang *et al*. [57] used Tn-Seq to identify sets of genes which the tubercle bacillus engages in order to survive host immunity - so-called ‘counteractomes’ - thereby uncovering a key role for *de novo* tryptophan biosynthesis in preventing the killing of *M. tuberculosis* by CD4 T cells.

Global phenotypic profiling has been powerfully reinforced by genome-wide transcriptional profiling of *M. tuberculosis* in various experimental models [59-61] and from clinical samples [62,63]. Initially, most analyses utilized DNA microarrays, but RNA sequencing (RNA-Seq) has now largely been adopted as the preferred technique [64-66]. A complex picture is emerging of the manner in which the bacillus modulates its transcriptome in response to environmental cues such as the stresses encountered in the phagosome [67] and the metabolic disturbances caused by chemical inhibition of cellular metabolism [68]. At a practical level, transcriptional profiling has provided a useful tool for categorizing the mechanisms of action of novel anti-mycobacterial agents [68,69]. From the broader drug discovery perspective, however, the results are ominous: the metabolic flexibility suggested by the various genome-wide transcriptional profiling studies indicates that *M. tuberculosis* is likely to be a difficult target for novel chemotherapies [6]. RNA-Seq has simultaneously uncovered an abundance of non-coding RNAs (ncRNAs) whose expression depends on both physiological stimuli and strain genetic background [41,64]. It seems likely, therefore, that these ncRNAs play a crucial role in the biology of TB infection, as suggested by recent work implicating the PhoP-dependent ncRNA, Mcr7, in regulating the secretion of a key mycobacterial antigen [70].

Comparing genome-wide essentiality and transcriptomic datasets has produced some surprising results: for example, very little overlap was found between the genes required for survival of *M. tuberculosis* in primary macrophages and those regulated by the intracellular environment, suggesting that gene expression screens may have limited value in identifying virulence genes in pathogens such as *M. tuberculosis* [49]. Nevertheless, the application of these and other genome-scale tools (for example, chromatin immunoprecipitation sequencing (ChIP-Seq) [70,71]), and their integration into systems biology approaches [59], promises to enable a systems-level understanding of the biology of *M. tuberculosis* as an exquisitely adapted human pathogen (Box 1). Importantly, concurrent advances in mycobacterial genetics, chemical biology, cell biology, and imaging have created a powerful platform for the development of novel anti-mycobacterial agents, as well as of diagnostics and biomarkers.

## Host responses to and biomarkers of *M. tuberculosis* infection

In a parallel approach, post-genomic tools have also been applied in analyzing the response of the human host to infection with *M. tuberculosis.* In a landmark study published in 2010, Ann O’Garra and colleagues [72] identified a 393-gene transcriptional signature in peripheral blood that was able to discriminate patients with active TB from the majority of latently infected and healthy controls. The neutrophil-driven interferon signature correlated with the extent of disease in those with active TB, as determined by chest X-ray [72], and diminished significantly after only two weeks on standard antitubercular therapy, reverting towards that observed in healthy controls [73]. The key findings of this study have been independently validated in different clinical settings and in diverse geographic locations [74-76]. More recently, gene expression signatures have also been identified that distinguish TB from other diseases prevalent in HIV-infected adults [77,78] and in children [79,80]. Together, these observations underscore the potential utility of blood transcriptional signatures as biomarkers for application in TB diagnosis and in monitoring of response to therapy.

Genomics research also promises to enable significant advances in the discovery of biomarkers and the development of point-of-care diagnostics. The elucidation of a blood transcriptional signature that can identify active TB cases [72] offers the possibility of significantly reducing the diagnostic delay that has been implicated in increased *M. tuberculosis* transmission and the emergence of drug resistance [81]. As noted elsewhere [82], the distinction between active TB and subclinical infection in this transcriptional assay is not absolute, which suggests that this test might be usefully applied to determine the extent of pathology (or bacterial burden) in latently infected individuals, and so might enable identification of those individuals most likely to progress to active disease. To our knowledge, the strength of the transcriptional signature has not been correlated with disease (or bacterial burden). It seems, therefore, that applying an equivalent assay in a non-human primate model [83] might enable calibration of the transcriptional signature against bacillary load and disease pathology. Whether a transcription-based assay of this nature can be applied in resource-limited, disease-endemic regions is currently uncertain; nevertheless, the diagnostic resolution enabled by such approaches suggests that further development is warranted. An additional consequence of these and other transcriptional analyses of host responses to *M. tuberculosis* infection is that fundamental questions have been raised about type I interferon signaling and its role in influencing the outcome of TB infection. As a result, the foundation has been established for systems immunology [84] approaches to understanding the immunopathogenesis of TB, and to developing vaccines and biomarkers through integration with mechanistic studies in cell-based and animal models of infection [85-87].

## Understanding the genotypic diversity of *M. tuberculosis* within and between hosts

Advances in high-throughput DNA-sequencing technology have transformed modern bacteriology [88], and their impact on TB genomics has been equally profound [89]. WGS of clinical *M. tuberculosis* isolates has enabled high-resolution insight into strain diversity [6,10], lineage-specific adaptation to host populations [11,12], and microvariation within hosts and communities [13-15]. In addition to providing strong evidence that bacillary genetics - and, therefore, function - are a significant element in determining the heterogeneous outcomes of infection, these observations suggest that WGS might be profitably incorporated into field trials of new-generation TB interventions, including drugs and vaccines. In one example, a retrospective observational study [90] that assessed patients from the REMoxTB trial of moxifloxacin-containing drug regimens [91] demonstrated the superiority of WGS over traditional genotyping methods for differentiating cases of relapse and re-infection. This study also confirmed a role for WGS in defining endpoints of clinical trials conducted in high-burden settings. In another example, recent work investigating the intra-patient evolution of *M. tuberculosis* in MDR patients undergoing longitudinal treatment demonstrated long-term co-existence of different bacillary sub-populations [92]. Notably, this study also documented the presence in individual patients of clonal sub-populations that possess different combinations of drug-resistance alleles, a result that has profound implications for phenotypic and molecular drug-resistance testing algorithms, which have traditionally assumed a monomorphic infecting *M. tuberculosis* population.

The growing evidence for genotypic diversity in *M. tuberculosis* impacts epidemiological analyses of strain prevalence and transmission, too. For example, a recent study has shown that the extent of genotypic diversity characterizing bacilli isolated from a single patient can be as great as that observed between samples obtained from patients along a transmission chain [93]. Consistent with earlier evidence from resected lungs [94] and sputum samples [95], the paper by Perez-Lago *et al*. [93] detected intra-patient diversity at both extrapulmonary and respiratory sites, which was interpreted as evidence that variability can be transmitted. As the authors suggest, this result raises important questions about the threshold that should be applied to differentiate relatedness among *M. tuberculosis* isolates for epidemiological analyses, and so renders the inference of transmission events inherently problematic.

To some extent, this difficulty is alleviated in low-incidence settings, especially where bacterial samples are accompanied by thorough clinical and epidemiological metadata. As an example, a retrospective observational study used WGS of archived samples to infer transmission directionality in household outbreaks of TB in the UK Midlands [96]. Again, the authors identified both intra-patient and between-host strain diversity, but the degree of variation was sufficiently limited to enable a framework to be established for the use of WGS data in field epidemiology. Importantly, these results suggested the possible use of WGS data to inform contact tracing, as well as to identify potential ‘super-spreaders’ - that is, *M. tuberculosis*-infected individuals who might be responsible for a disproportionate number of secondary cases. Even though high-burden settings are likely to pose a special challenge to the application of genomic epidemiology, there is evidence to support the potential of high-resolution genotyping in defining transmission chains independent of drug resistance [97]. This study from China appears to be the only one of its kind to date in a TB endemic region, but it does suggest the utility of genomic epidemiology, especially where augmented by good clinical, demographic, and social data [98].

## Challenges and perspectives

As an obligate pathogen, *M. tuberculosis* is distinguished from many other infectious organisms (bacterial, viral, and parasitic) which have recourse to non-human reservoirs. Nevertheless, the application of modern genomics techniques in these diverse systems reinforces the potential to elucidate functions and properties that are essential to pathogenesis [99], or which drive the rapid emergence of outbreak strains [100] and ensure their long-term circulation within host populations [101]. High-resolution genotyping, in particular, has revealed that the diversification of clonal infecting strains into ‘clouds of diversity’ [88] is a feature of many different pathogenic organisms. Determining the extent to which intraspecific diversity is crucial for pathogenesis therefore represents a key research question, and will require the development of systems biology approaches to determine the emergent properties of microdiverse infecting populations.

For TB, it will be useful to consider the immediate research priorities in the context of the major lifecycle stages - active disease, clinical latency, and transmission - and to prioritize genomics applications that are most likely to inform future drug and vaccine development programs (Box 2). The application of advanced ’omic tools is key to novel approaches such as systems epidemiology [102] that aim to combine high-resolution epidemiological data with systems biology. Nevertheless these techniques must also be harnessed in developing methods for predictive epidemiology that can enable genuinely transformative interventions in TB incidence. As outlined above, the use of WGS to enable definitive differentiation of relapse from re-infection has very significant implications for trials of experimental drug regimens [90]. This is a particularly important consideration in high-burden settings where the force of infection is elevated [2], mixed infections common [103], and a large percentage of recurrent TB is due to exogenous re-infection [104]. Moreover, the potential for epigenetic modifications, such as DNA methylation, to alter bacillary physiology [105] suggests that novel sampling methods and sequencing technologies [100] will be useful in determining the spectrum of physiological states adopted by *M. tuberculosis* within the host and which might impact drug efficacy. Similarly, establishing whether prior infection with one bacillary genotype might predispose to re-infection with a separate genotype following chemotherapeutic elimination [90] is essential, not only for control programs but also for TB vaccine development strategies.

In summary, genomics research will continue to drive efforts to understanding the evolutionary processes that have enabled the adaptation of *M. tuberculosis* as a human pathogen. Translating the exciting advances provided by genomics into new tools that can radically transform TB control will require significant and sustained resourcing. It is incumbent upon the TB research community to ensure that there is sufficient political will to make this happen.

## Box 1. Towards systems biology for tuberculosis

**A definition of systems biology**

**The term 'systems biology' is generally used to describe the interacting components of a biological system. Through iterative testing and validation, a mathematical model of the system is constructed, modified, and re-constructed using experimental data obtained from diverse sources. These sources are primarily** ’**omics applications such as genomics, transcriptomics, proteomics and metabolomics, but also include ‘classic’ approaches such as molecular biology, genetics, and microbiology. Critically, the model must be able to predict the emergent properties of the system, as well as the impact on the system of external factors and stimuli that might alter specific components or groups of components.**

***Systems biology of TB***

**The lifecycle of*M. tuberculosis*is driven entirely within the context of human infection: transmission from an infected individual, infection of a new recipient, development of active disease or establishment of a clinically latent state that is able to reactivate, and transmission to a new host. As a result, TB as a disease within an individual might be considered an emergent property of multiple interactions that occur over a range of timescales and at different levels - anatomical, cellular, and molecular - all of which involve elements derived from both bacillus and host. At the level of host populations, systems epidemiology seeks to elucidate the factors - demographic, social, and systemic - that enable the propagation of select*M. tuberculosis*lineages and mutants that are able to survive in the face of control programs and in competition with other genotypes.**

***Some approaches that might be adopted***

**Direct investigations of mycobacterial function in the context of the complete biological system - the*M. tuberculosis*-infected host - are rare, but will be crucial if the barriers to translating the observations from basic research into practical outcomes are to be overcome. A suite of** ’**omics techniques must be applied to clinical samples to capture the full diversity of metabolic, proteomic, transcriptomic, and genomic features that characterize the diversity of potentially heterogeneous mycobacterial populations within discrete host compartments and anatomical loci. For example:**

• **Comparative genomics could be used to identify evidence of convergent evolution in clinical*M. tuberculosis*isolates - both independent of, and associated with, drug resistance.**

• **Combining and comparing genotypic, epigenetic, and phenotypic data from bacilli captured at different stages of infection - for example, aerosol-encapsulated organisms released by individuals who have active TB versus sputum-based organisms induced for standard clinical diagnostics, paucibacillary populations in immunologically inactive lesions versus bacilli obtained from TB pneumonia, and so on. In all cases, these data should be overlayed with the diversity of host cellular and immunopathological phenotypes.**

• **Corresponding data should be obtained from experimental models in order to identify the disease-relevant phenotypes and functional interactions that each model system is best able to recapitulate.**

## Box 2. Translational priorities

***Identifying and intervening in transmission chains***

**Can we develop WGS-based methods to identify transmission ‘hotspots’ and transmission chains to enable real-time interventions to limit the spread of virulent and/or drug-resistant strains?**

***Identifying the factors that impact infection outcomes***

**Can we apply systems biology methods to determine the bacillary and host genetic factors that drive disease progression in specific individuals?**

***Drug treatment***

**Can we utilize WGS-based methods to identify mixed*M. tuberculosis*infections prior to initiation of treatment?**

**Can we exploit host transcriptional profiling to determine the response to treatment?**

***Latent infection and vaccinology***

**Can we use host transcriptional profiling to identify (and treat) latently infected individuals with a high probability of progressing to active disease?**

**Can knowledge about mycobacterial diversity be used to guide vaccine development and use in TB-endemic regions?**

***Mycobacterial population biology and genomics***

**Can we determine the impact of intraspecific diversity on disease progression and the emergence of drug resistance?**

## Abbreviations

ChIP-Seq: Chromatin immunoprecipitation sequencing
HGT: Horizontal gene transfer
MDR: Multidrug resistant
MTBC: *Mycobacterium tuberculosis* complex
ncRNA: Non-coding RNA
PAT: Polyacyltrehalose
RNA-Seq: RNA sequencing
SL: Sulfolipid
SNP: Single nucleotide polymorphism
TB: Tuberculosis
Tn: Transposon
Tn-Seq: Transposon sequencing
TraSH: Transposon site hybridization
WGS: Whole-genome sequencing
XDR: Extensively drug resistant
